# The Class I Histone Deacetylase Inhibitor MS-275 Prevents Pancreatic Beta Cell Death Induced by Palmitate

**DOI:** 10.1155/2014/195739

**Published:** 2014-12-31

**Authors:** Valérie Plaisance, Laure Rolland, Valéry Gmyr, Jean-Sébastien Annicotte, Julie Kerr-Conte, François Pattou, Amar Abderrahmani

**Affiliations:** ^1^European Genomic Institute for Diabetes (EGID) FR 3508, University of Lille, CNRS UMR 8199, and Faculty of Medicine West, 1 Place de Verdun, 59045 Lille, France; ^2^Department of Endocrine Surgery, Lille University Hospital, INSERM UMR 859, University of Lille, EGID FR 3508, Biotherapies for Diabetes, Lille, France

## Abstract

Elevation of the dietary saturated fatty acid palmitate contributes to the reduction of functional beta cell mass in the pathogenesis of type 2 diabetes. The diabetogenic effect of palmitate is achieved by increasing beta cell death through induction of the endoplasmic reticulum (ER) stress markers including activating transcription factor 3 (Atf3) and CAAT/enhancer-binding protein homologous protein-10 (Chop). In this study, we investigated whether treatment of beta cells with the MS-275, a HDAC1 and HDAC3 activity inhibitor which prevents beta cell death elicited by cytokines, is beneficial for combating beta cell dysfunction caused by palmitate. We show that culture of isolated human islets and MIN6 cells with MS-275 reduced apoptosis evoked by palmitate. The protective effect of MS-275 was associated with the attenuation of the expression of Atf3 and Chop. Silencing of HDAC3, but not of HDAC1, mimicked the effects of MS-275 on the expression of the two ER stress markers and apoptosis. These data point to HDAC3 as a potential drug target for preserving beta cells against lipotoxicity in diabetes.

## 1. Introduction

Type 2 diabetes arises when beta cells produce insufficient insulin to meet the increased hormone demand, caused by insulin resistance. Impaired insulin plasma level is the consequence of reduced capacity for secreting insulin in response to nutrients and insufficient beta cells number. Lifestyle changes together with excessive visceral adiposity and genetic factors predispose to the diabetes risk, and thereby to beta cell dysfunction [1, 2]. These factors promote low chronic grade inflammation, which affects beta cell function and mass [3]. Several reports have shown that treatment of beta cells with histone deacetylase (HDAC) inhibitors can prevent the adverse effects of cytokines [4, 5]. These inhibitors include the HDAC1 and HDAC3 MS-275 compound also called entinostat [4, 5]. The latter is undergoing clinical trials for treatment of cancers including breast, lymphoma, and lung [6]. Coexposure of islets and beta cell line to the MS-275 prevents death caused by cytokines [5]. The protective effect of MS-275 relies on HDAC3 [5]. Silencing of HDAC3 mimics the effect of MS-275 against beta cell death [5].

Chronic elevation of saturated free fatty acids may be the link between visceral adiposity and low grade inflammation in type 2 diabetes [7–9]. Numerous studies underline the diabetogenic effect of palmitate in eliciting beta cell death in the pathogenesis of type 2 diabetes [9–17]. The harmful effects of palmitate are achieved by activation of some important signalling pathways, including activation of endoplasmic reticulum (ER) stress [18–22]. Activation of ER stress triggers the unfolded protein response (UPR) [23, 24]. In response to prolonged exposure to palmitate, UPR promotes the expression of CAAT/enhancer-binding protein homologous protein-10 (CHOP, also known as the DNA-damage-inducible transcription factor 3) and activates transcription factor 3 (ATF3), thus leading to apoptosis [25, 26]. Changes in CHOP and ATF3 expression have been associated with beta cell dysfunction in diabetes [22, 27–30]. In this study, we investigated the effects of MS-275 on the adverse effects evoked by palmitate.

## 2. Materials and Methods

### 2.1. Materials

The saturated fatty acid palmitate (sodium salts, Sigma Aldrich, St. Louis, MO) was coupled to bovine serum albumin-fatty acid free by 1 h agitation at 37°C and freshly prepared for each experiment [31]. This procedure yielded BSA-coupled fatty acids in a molar ratio of 5 : 1. The MS-275 was purchased from Sigma-Aldrich (St. Louis, MO). The antibodies against Chop, Atf3, TATA box binding protein (Tbp), and HDAC1 were obtained from Santa Cruz Biotechnology (CA, USA). The anti-HDAC3 and anti-*β*-actin antibodies were from Cell Signaling Technology (MA, USA) and Sigma (Saint Quentin, France), respectively.

### 2.2. Cell Culture and Transfection

The mouse insulin-secreting cell line MIN6 was cultured exactly as previously described [32]. The siRNA duplexes directed against HDAC1 (si-HDAC1), HDAC3 (si-HDAC3), and GFP (si-GFP) were introduced using the Lipofectamine 2000 (Invitrogen AG) exactly as described [33]. Human pancreases were harvested from adult brain-dead donors in accordance with French regulations and with the local Institutional Ethical Committee from the “Centre Hospitalier Régional et Universitaire de Lille.” Pancreatic islets were isolated after ductal distension of the pancreas and digestion of the tissue as described previously [34]. All experiments were carried out at least on islets cells of >80% purity. Purified islets were cultured in CMRL 1066 medium (Gibco BRL, Life Technologies) containing 0.625% free fatty acid HSA (Roche Diagnostics), penicillin (100 *μ*UI/mL), and streptomycin (100 *μ*g/mL). A pool of 4 siRNAs was used to knock down HDAC1 and HDAC3 expression (ON-TARGETplus SMARTpool, Thermo Scientific Dharmacon).

### 2.3. Quantitative PCR

Total RNA was extracted using guanidinium thiocyanate-phenol-chloroform and converted to cDNA as described [35]. Real-time quantitative PCR assays were carried out on the Bio-Rad MyiQ real-time PCR detection system using iQ SyBr Green Supermix (Bio-Rad) as the amplification system with 100 nM primers and 2 *μ*L of template (RT product) in 20 *μ*L of PCR volume and annealing temperature of 59°C. Primers sequences were human* ATF3*; forward 5′-CTCCTGGGTCACTGGTGTTT-3′ and reverse 5′-GTTCTCTGCTGCTGGGATTC-3′; mouse* Atf3*; forward 5′-AAGACAGAGTGCCTGCAGAA-3′ and reverse 5′-GTGCCACCTCTGCTTAGCTC-3′; human* CHOP* forward 5′-GTGAATCTGCACCAAGCATGA-3′ and reverse 5′-AAGGTGGGTAGTGTGGCCC-3′; mouse* Chop* forward 5′-TTCACTACTCTTGACCCTGCGT-3′ and reverse 5′-CACTGACCACTCTGTTTCCGTTTC-3′; human and mouse* Rplp0*/*RPLP0* forward 5′-ACCTCCTTTTTCCAGGCTTT-3′ and reverse 5′-CCCACTTTGTCTCCAGTCTTG-3′.

### 2.4. Western Blotting

Nuclear protein extracts from cells were prepared exactly as previously described [16]. For western blotting experiments, 25–40 *μ*g of protein extracts was separated on 10% SDS-polyacrylamide gel and electrically blotted to nitrocellulose membrane. The proteins were detected after an overnight incubation of the membrane at 4°C with the specific primary antibodies against HDAC1 (dilution 1 : 1000), Tata box binding protein (Tbp, dilution 1 : 1000), *β*-actin (dilution 1 : 5000), HDAC3 (dilution 1 : 1000), and Chop (dilution 1 : 500) in buffer containing 0.1% Tween 20 with either 5% milk (for HDAC1, Chop, *β*-actin, and Tbp) or 5% BSA (for HDAC3 and Atf3). Proteins were visualized with IRDye800 or IRDye700 (Eurobio, Les Ulis, France) as secondary antibodies. Quantification was performed using the Odyssey infrared imaging system (Eurobio).

### 2.5. Apoptosis Assay

Apoptosis was determined by determining mono- and oligonucleosomes in the cytoplasmic fraction by ELISA kit (Roche Molecular Biochemicals) and by scoring cells displaying pyknotic or fragmented nuclei (visualized with Hoechst 33342) [36]. The counting was performed blind by two different experimenters.

### 2.6. Statistical Analysis

ANOVA was used for statistical significance, followed by the post hoc Bonferroni test (Dunnett's test) when experiments included more than two groups.

## 3. Results

### 3.1. MS-275 Antagonizes the Deleterious Effects of Palmitate in MIN6 and Isolated Human Islets

**Figure 1:**
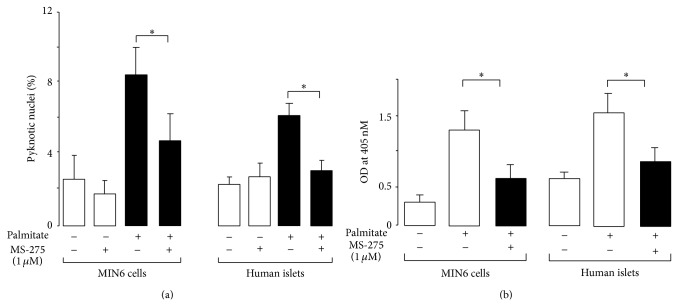
MS-275 alleviates apoptosis caused by palmitate in isolated human islets and MIN6 cells. Effect of MS-275 on (a) the number of pyknotic nuclei and (b) histone-associated DNA fragments. MIN6 or dispersed human islets cells were cultured with 0.5 mM palmitate (Palm, Sigma Aldrich, St. Louis, MO) or bovine serum albumin (BSA; −) with 1 μM MS-275 (Sigma Aldrich, St. Louis, MO) or vehicle (DMSO; −) for 48 hrs. The data are the mean ± SD of 4 independent experiments (^*^ *P* < 0.05).

Previous studies including ours have found that palmitate increases death in different insulin-secreting cells including MIN6 cells and isolated human islets cultured with palmitate for 48 hrs [13, 16, 18, 20, 37]. Palmitate triggers some adverse effects under normal glucose concentration in human and mouse beta cells [13, 38]. We confirmed that exposure of MIN6 and isolated human islets cells to 0.5 mM palmitate for 48 hrs caused a 3- and 4-fold increase in apoptosis, respectively (Figure 1). Different concentrations of MS-275 have been previously tested in insulin-secreting cells [5]. Preliminary studies showed that concentrations of MS-275 above 1 *μ*M were deleterious for cell viability (data not shown). However culture of MIN6 cells with 1 *μ*M MS-275 did not affect cell survival under normal culture condition (Figure 1(a)) whereas, as expected, it caused a 30–40% significant reduction in the total HDAC activity (Supplementary Figure 1(a) available online at http://dx.doi.org/10.1155/2014/195739). A previous study reports that prolonged exposure of insulin-producing cells to palmitate did not change total HDAC activity [39]. In line with this observation, chronic culture of MIN6 cells with the saturated fatty acid did affect neither total HDAC activity nor HDAC1 and HDAC3 mRNA levels (Supplementary Figures 1(a) and 1(b)). In fact, we found that the drop of HDAC activity caused by the MS-275 was associated with an increase in survival of MIN6 and isolated human islets cells in response to palmitate (Figures 1(a) and 1(b)). While chronic exposure of MIN6 cells to palmitate reduces preproinsulin mRNA level [16], we confirmed that the lipid did not affect the hormone mRNA level in isolated human islets (Supplementary Figure 2(a)) as previously described [40]. However, insulin content is diminished in islets from different species and MIN6 cells chronically exposed to palmitate [40–42]. The MS-275 improved the preproinsulin mRNA (Supplementary Figure 2(a)) and insulin content (Supplementary Figure 2(b)) of cells chronically cultured with palmitate. Palmitate impairs glucose-induced insulin secretion [16]. However, the MS-275 was insufficient for antagonizing the harmful effect of the lipid on glucose-induced insulin secretion in MIN6 cells (Supplementary Figure 2(c)). All these data indicate that the cytoprotective effect of MS-275 is associated with an improved insulin expression.

**Figure 2:**
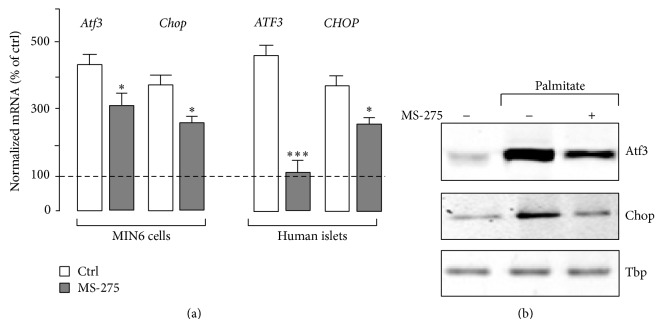
Effects of MS-275 on the expression the ER stress markers. (a) The mRNA of* Atf3/ATF3 *and* Chop/CHOP *was quantified by quantitative real-time PCR from MIN6 cells and isolated human islets cultured with 0.5 mM palmitate plus MS-275 (*grey bars*) or DMSO (*open bars*) for 48 hrs. The mRNA levels were normalized against the* Rplp0/RPLP0* and the expression levels from cells cultured with BSA (−) were set to 100%. Data are the mean of ± SEM of 3 independent experiments (^***^ *P* < 0.001; ^*^ *P* < 0.05). (b) For western blotting analysis, nuclear proteins were prepared from cells cultured for 48 hrs with 0.5 mM palmitate plus DMSO (−) or 1 μM MS-275. Immunoblotting was done using the anti-Atf3, anti-Chop, and anti-Tbp as the control. The figure shows the result of a representative experiment out of three. The data are the mean ± SEM of 4 independent experiments (^*^ *P* < 0.05).

Elevation of ATF3 and CHOP contributes to the UPR-induced death caused by palmitate [37]. We next investigated whether the protective effect triggered by MS-275 is associated with reduced level of the two ER stress markers. Quantitative PCR showed that MS-275 attenuated induction of* Atf3*/*ATF3* and* Chop*/*CHOP* by palmitate in MIN6 cells and human islets (Figure 2(a)). Western blotting experiments confirmed the antagonist effects of MS-275 on the increase of Atf3 and Chop evoked by palmitate (Figure 2(b)).

### 3.2. Silencing of HDAC3 Mimics the Effects of MS-275

**Figure 3:**
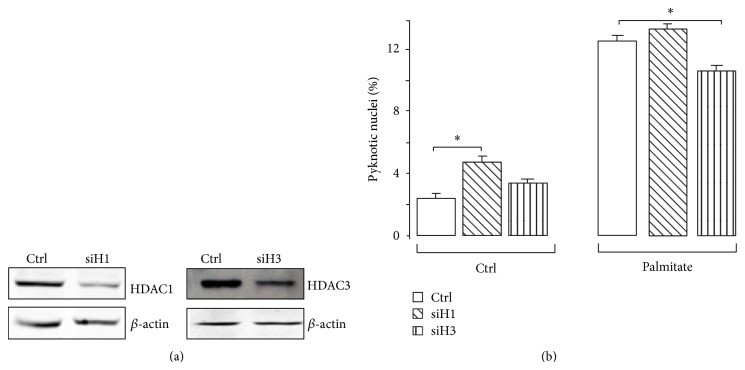
Impact of HDAC1 and HDAC3 silencing on apoptosis evoked by palmitate. (a) Western blotting analysis of HDAC1 and HDAC3 abundance and (b) measurement of apoptosis upon silencing of HDAC1 and HDAC3 in MIN6 cells. Cells were transfected with the siRNAs directed against either HDAC1 (siH1), HDAC3 (siH3), or GFP (ctrl). Palmitate was added 24 hrs after transfection. Nuclear proteins were prepared and pyknotic nuclei were counted 48 hrs later. The immunoblotting was achieved using the anti-HDAC1, anti-HDAC3, and anti-β-actin antibodies. The figure shows the result of a representative experiment out of three.

**Figure 4:**
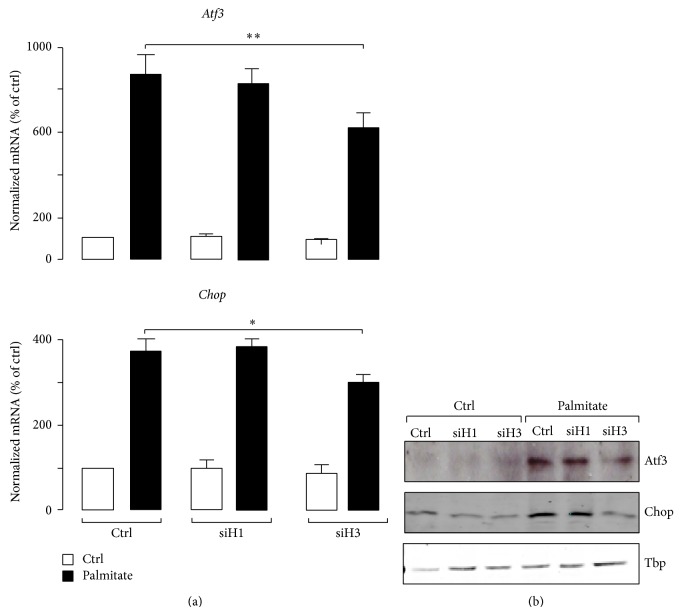
Effects of HDAC1 and HDAC3 silencing on the ER stress markers. The Atf3 and Chop level of MIN6 cells transfected with siH1, siH3, or control siRNAs (siGFP) was quantified by (a) quantitative PCR and (b) western blotting experiments. Cells were cultured with palmitate (*filled bars*) or BSA (*open bars*) 24 hrs after transfection. Total RNA and nuclear protein extracts were prepared 48 hrs later. The mRNA levels were normalized against the* Rplp0 *and the expression levels from cells cultured with BSA (−) and transfected with siGFP were set to 100%. Data are the mean of ± SEM of 3 independent experiments (^**^ *P* < 0.01; ^*^ *P* < 0.05). For western blotting analysis, the anti-Atf3, anti-Chop, and anti-Tbp as the control were used. The figure shows the result of a representative experiment out of three.

MS-275 is a class I HDAC inhibitor that selectively inhibits HDAC1 and HDAC3 activities [43]. Silencing of HDAC1 and HDAC3 by siRNA duplexes (siH1 and siH3) was performed to determine which of the two HDACs was involved in the effect of MS-275. Western blotting experiments confirmed the efficiency of the two siRNA duplexes for reducing the HDAC1 and HDAC3 abundances in MIN6 cells (Figure 3(a)). While the decrease of HDAC1 did not protect MIN6 cells against apoptosis caused by palmitate, suppression of HDAC3 did (Figure 3(b)). In addition, siH3, but not siH1, mimicked the effect of MS-275 by alleviating the elevation of Atf3 and Chop mRNA and protein levels provoked by the fatty acid (Figures 4(a) and 4(b)), suggesting a role for HDAC3 as the target of MS-275 for triggering the protective effect.

## 4. Discussion

The saturated fatty acid palmitate is deemed to be an important diabetogenic factor that links obesity, insulin resistance, and reduced functional beta cell mass [2, 9]. One of the harmful effects triggered by palmitate on beta cells is the reduction of cell survival [19, 20, 37]. This is in part achieved by inducing the expression of Chop and Atf3 through UPR [25, 26]. Herein, we show that MS-275 prevents the increase of the two transcription factors and apoptosis caused by palmitate. Silencing of HDAC3, but not HDAC1, mimicked the effects of the compound. Palmitate did not impinge the HDAC3 expression, supporting a role for the lipid in triggering the activity of this Hdac. Similar to most HDACs, HDAC3 binds to promoters as a corepressor [44]. HDAC3 activity produces some changes in the chromatin structure through histone deacetylation, leading to silencing of gene expression [44]. Based on this function, a direct binding of HDAC3 to the* Chop/CHOP* and* Atf3/ATF3* promoters in response to palmitate seems unlikely. The most likely scenario is that HDAC3 directly regulates the expression of negative regulatory factor such as transcriptional repressor(s) or microRNAs. Reduced activity of these negative regulators by HDAC3 may elevate the Chop/CHOP and Atf3/ATF3 mRNA and protein levels in response to palmitate, thus leading to apoptosis. Inversely, MS-275 or silencing of HDAC3 may prevent the silencing of the negative regulators caused by palmitate. The consequence of such derepression would lead to reduction of Chop/CHOP and Atf3/ATF3 mRNA and protein expression. Future experiments plan to identify the repressor(s) through which HDAC3 controls the elevation of the two ER stress markers level and apoptosis caused by lipotoxicity.

There is increasing evidence supporting the therapeutic use of HDAC inhibition as novel drugs for neurodegenerative and other inflammatory diseases [45]. At present, a growing number of reports indicate beneficial effects of HDAC inhibitors in metabolic diseases. Treatment with the pan-HDACs inhibitors sodium butyrate or the class I HDAC inhibitor MS-275 improves insulin sensitivity in mice with diet-induced obesity [46] and obese db/db mice [47], respectively. A protective role of class I HDAC inhibition against beta cell apoptosis and dysfunction elicited by cytokines has been further reported [5, 48], thus underlining the potential interest of HDAC inhibition for diabetes care. In this regard, HDAC3 has been suggested as an antidiabetic drug target [49]. In conclusion, in this study we provide additional evidence that HDAC3 could also be a potential drug target for preserving pancreatic beta cells against apoptosis induced by lipotoxicity in type 2 diabetes.

## Supplementary Material

Supplementary Figure 1: Effect of palmitate on the HDACs activity and expression.
Supplementary Figure 2: Measurement of preproinsulin mRNA, insulin content and glucose-induced insulin secretion in response to MS-275.

## References

[1] Bonnefond A., Froguel P., Vaxillaire M. (2010). The emerging genetics of type 2 diabetes. *Trends in Molecular Medicine*.

[2] Prentki M., Nolan C. J. (2006). Islet *β* cell failure in type 2 diabetes. *The Journal of Clinical Investigation*.

[3] Donath M. Y., Böni-Schnetzler M., Ellingsgaard H., Halban P. A., Ehses J. A. (2010). Cytokine production by islets in health and diabetes: Cellular origin, regulation and function. *Trends in Endocrinology and Metabolism*.

[4] Lundh M., Christensen D. P., Damgaard Nielsen M. (2012). Histone deacetylases 1 and 3 but not 2 mediate cytokine-induced beta cell apoptosis in INS-1 cells and dispersed primary islets from rats and are differentially regulated in the islets of type 1 diabetic children. *Diabetologia*.

[5] Chou D. H.-C., Holson E. B., Wagner F. F. (2012). Inhibition of histone deacetylase 3 protects beta cells from cytokine-induced apoptosis. *Chemistry & Biology*.

[6] Knipstein J., Gore L. (2011). Entinostat for treatment of solid tumors and hematologic malignancies. *Expert Opinion on Investigational Drugs*.

[7] Lin X., Song K., Lim N. (2009). Risk prediction of prevalent diabetes in a Swiss population using a weighted genetic score-the CoLaus Study. *Diabetologia*.

[8] Paolisso G., Tataranni P. A., Foley J. E., Bogardus C., Howard B. V., Ravussin E. (1995). A high concentration of fasting plasma non-esterified fatty acids is a risk factor for the development of NIDDM. *Diabetologia*.

[9] Eguchi K., Manabe I., Oishi-Tanaka Y. (2012). Saturated fatty acid and TLR signaling link *β* cell dysfunction and islet inflammation. *Cell Metabolism*.

[10] Meier J. J., Breuer T. G. K., Bonadonna R. C. (2012). Pancreatic diabetes manifests when beta cell area declines by approximately 65% in humans. *Diabetologia*.

[11] Butler A. E., Janson J., Bonner-Weir S., Ritzel R., Rizza R. A., Butler P. C. (2003). *β*-cell deficit and increased *β*-cell apoptosis in humans with type 2 diabetes. *Diabetes*.

[12] Donath M. Y., Schumann D. M., Faulenbach M., Ellingsgaard H., Perren A., Ehses J. A. (2008). Islet inflammation in type 2 diabetes: from metabolic stress to therapy. *Diabetes Care*.

[13] Dubois M., Kerr-Conte J., Gmyr V. (2004). Non-esterified fatty acids are deleterious for human pancreatic islet function at physiological glucose concentration. *Diabetologia*.

[14] Hagman D. K., Hays L. B., Parazzoli S. D., Poitout V. (2005). Palmitate inhibits insulin gene expression by altering PDX-1 nuclear localization and reducing MafA expression in isolated rat islets of Langerhans. *Journal of Biological Chemistry*.

[15] Poitout V., Robertson R. P. (2002). Minireview: secondary *β*-cell failure in type 2 diabetes—a convergence of glucotoxicity and lipotoxicity. *Endocrinology*.

[16] Plaisance V., Perret V., Favre D. (2009). Role of the transcriptional factor C/EBP*β* in free fatty acid-elicited *β*-cell failure. *Molecular and Cellular Endocrinology*.

[17] Kashyap S., Belfort R., Gastaldelli A. (2003). A sustained increase in plasma free fatty acids impairs insulin secretion in nondiabetic subjects genetically predisposed to develop type 2 diabetes. *Diabetes*.

[18] Watson M. L., Macrae K., Marley A. E., Hundal H. S. (2011). Chronic effects of palmitate overload on nutrient-induced insulin secretion and autocrine signalling in pancreatic MIN6 beta cells. *PLoS ONE*.

[19] Cnop M., Ladrière L., Igoillo-Esteve M., Moura R. F., Cunha D. A. (2010). Causes and cures for endoplasmic reticulum stress in lipotoxic *β*-cell dysfunction. *Diabetes, Obesity and Metabolism*.

[20] Akerfeldt M. C., Howes J., Chan J. Y. (2008). Cytokine-Induced *β*-cell death is independent of endoplasmic reticulum stress signaling. *Diabetes*.

[21] Kharroubi I., Ladrière L., Cardozo A. K., Dogusan Z., Cnop M., Eizirik D. L. (2004). Free fatty acids and cytokines induce pancreatic *β*-cell apoptosis by different mechanisms: role of nuclear factor-*κ*B and endoplasmic reticulum stress. *Endocrinology*.

[22] Laybutt D. R., Preston A. M., Åkerfeldt M. C. (2007). Endoplasmic reticulum stress contributes to beta cell apoptosis in type 2 diabetes. *Diabetologia*.

[23] Eizirik D. L., Cardozo A. K., Cnop M. (2008). The role for endoplasmic reticulum stress in diabetes mellitus. *Endocrine Reviews*.

[24] Shi Y., Taylor S. I., Tan S.-L., Sonenberg N. (2003). When translation meets metabolism: multiple links to diabetes. *Endocrine Reviews*.

[25] Ron D., Walter P. (2007). Signal integration in the endoplasmic reticulum unfolded protein response. *Nature Reviews Molecular Cell Biology*.

[26] Jiang H.-Y., Wek S. A., McGrath B. C. (2004). Activating transcription factor 3 is integral to the eukaryotic initiation factor 2 kinase stress response. *Molecular and Cellular Biology*.

[27] Hartman M. G., Lu D., Kim M.-L. (2004). Role for activating transcription factor 3 in stress-induced *β*-cell apoptosis. *Molecular and Cellular Biology*.

[28] Marchetti P., Bugliani M., Lupi R. (2007). The endoplasmic reticulum in pancreatic beta cells of type 2 diabetes patients. *Diabetologia*.

[29] Zmuda E. J., Qi L., Zhu M. X., Mirmira R. G., Montminy M. R., Hai T. (2010). The roles of ATF3, an adaptive-response gene, in high-fat-diet-induced diabetes and pancreatic *β*-cell dysfunction. *Molecular Endocrinology*.

[30] Oyadomari S., Koizumi A., Takeda K. (2002). Targeted disruption of the Chop gene delays endoplasmic reticulum stress-mediated diabetes. *Journal of Clinical Investigation*.

[31] Busch A. K., Cordery D., Denyer G. S., Biden T. J. (2002). Expression profiling of palmitate- and oleate-regulated genes provides novel insights into the effects of chronic lipid exposure on pancreatic *β*-cell function. *Diabetes*.

[32] Lilla V., Webb G., Rickenbach K. (2003). Differential gene expression in well-regulated and dysregulated pancreatic *β*-cell (MIN6) sublines. *Endocrinology*.

[33] Ferdaoussi M., Abdelli S., Yang J.-Y. (2008). Exendin-4 protects *β*-cells from interleukin-1*β*-induced apoptosis by interfering with the c-Jun NH2-terminal kinase pathway. *Diabetes*.

[34] Vantyghem M.-C., Kerr-Conte J., Arnalsteen L. (2009). Primary graft function, metabolic control, and graft survival after islet transplantation. *Diabetes Care*.

[35] Favre D., Le Gouill E., Fahmi D. (2011). Impaired expression of the inducible cAMP early repressor accounts for sustained adipose CREB activity in obesity. *Diabetes*.

[36] Ezanno H., Pawlowski V., Abdelli S. (2014). JNK3 is required for the cytoprotective effect of exendin 4. *Journal of Diabetes Research*.

[37] Cunha D. A., Hekerman P., Ladrière L. (2008). Initiation and execution of lipotoxic ER stress in pancreatic *β*-cells. *Journal of Cell Science*.

[38] Sargsyan E., Bergsten P. (2011). Lipotoxicity is glucose-dependent in INS-1E cells but not in human islets and MIN6 cells. *Lipids in Health and Disease*.

[39] Malmgren S., Spégel P., Danielsson A. P. H. (2013). Coordinate changes in histone modifications, mRNA levels, and metabolite profiles in clonal INS-1 832/13 *β*-cells accompany functional adaptations to lipotoxicity. *The Journal of Biological Chemistry*.

[40] Fred R. G., Bang-Berthelsen C. H., Mandrup-Poulsen T., Grunnet L. G., Welsh N. (2010). High glucose suppresses human islet insulin biosynthesis by inducing miR-133a leading to decreased polypyrimidine tract binding protein-expression. *PLoS ONE*.

[41] Kato T., Shimano H., Yamamoto T. (2008). Palmitate impairs and eicosapentaenoate restores insulin secretion through regulation of SREBP-1c in pancreatic islets. *Diabetes*.

[42] Iizuka K., Nakajima H., Namba M. (2002). Metabolic consequence of long-term exposure of pancreatic *β* cells to free fatty acid with special reference to glucose insensitivity. *Biochimica et Biophysica Acta—Molecular Basis of Disease*.

[43] Hu E., Dul E., Sung C.-M. (2003). Identification of novel isoform-selective inhibitors within class I histone deacetylases. *Journal of Pharmacology and Experimental Therapeutics*.

[44] Karagianni P., Wong J. (2007). HDAC3: taking the SMRT-N-CoRrect road to repression. *Oncogene*.

[45] Christensen D. P., Dahllöf M., Lundh M. (2011). Histone deacetylase (HDAC) inhibition as a novel treatment for diabetes mellitus. *Molecular Medicine*.

[46] Gao Z., Yin J., Zhang J. (2009). Butyrate improves insulin sensitivity and increases energy expenditure in mice. *Diabetes*.

[47] Galmozzi A., Mitro N., Ferrari A. (2013). Inhibition of class i histone deacetylases unveils a mitochondrial signature and enhances oxidative metabolism in skeletal muscle and adipose tissue. *Diabetes*.

[48] Larsen L., Tonnesen M., Ronn S. G. (2007). Inhibition of histone deacetylases prevents cytokine-induced toxicity in beta cells. *Diabetologia*.

[49] Meier B. C., Wagner B. K. (2014). Inhibition of HDAC3 as a strategy for developing novel diabetes therapeutics. *Epigenomics*.

