# Supplementary material for: The Class I Histone Deacetylase Inhibitor MS-275 Prevents Pancreatic Beta Cell Death Induced by Palmitate
Source: J Diabetes Res. 2014 Dec 31;2014:195739. doi: 10.1155/2014/195739 (PMC4294305; doi:10.1155/2014/195739)

Supplementary Figure 1: Effect of palmitate on the HDACs activity and expression. **a**) Measurement of HDAC activity in response to plamitate and MS-275. Total proteins were prepared from MIN6 cells that were cultured with (*filled bars*) or without (*open bars*, BSA) 0.5 mM palmitate. The cells co-cultured with either MS-275 or DMSO (-). HDACs activity was measured by direct colorimetric assay kit (Epigentek). Data are the mean ± SEM of 3 independent experiments (*, p<0,05). HDACs activity from control cells (open bar) was set as 100%. **b**) Quantification of *Hdac1* and *Hdac3* mRNA levels in response to palmitate. The *Hdac1* and *Hdac3* mRNA levels were determined by quantitative real-time PCR in MIN6 cells that were cultured with (*filled bars*) or without (*open bars*, BSA) 0.5 mM palmitate. The primer sequence were the followings: mouse *Hdac1*; Forward: 5'-AGTCTGTTACTACTACGACGGG-3', and Reverse: 5'-TGAGCAGCAAATTGTGAGTCAT-3'; mouse *Hdac3;* Forward: 5'- GGTTCAGGAAGCCTTCTACCT-3', and Reverse: 5'-ACTCTCTGCTCCAACTTCATACA-3'. The mRNA levels were normalized against *Rplp0* and the results obtained in cells cultured with vehicle were set to 100%. Data are the mean of ± SEM of 3 independent experiments.

a

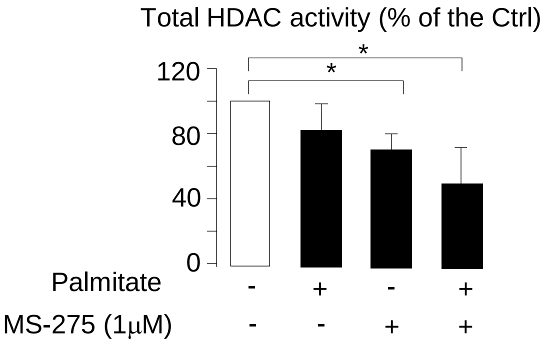

b

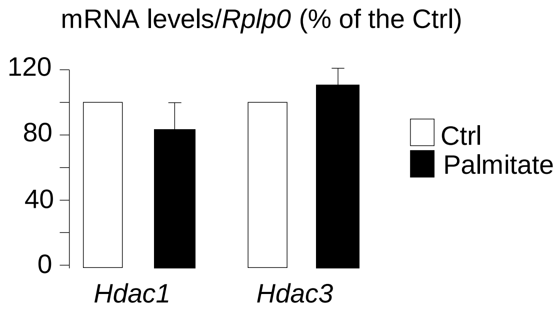

Supplementary Figure 2: Measurement of preproinsulin mRNA, insulin content and glucose-induced insulin secretion in response to MS-275. a) quantification of preproinsulin mRNA by PCR**.** MIN6 or human islets were co-incubated with (filled bars) or without (open bars) 0.5 mM palmitate for 48h in the presence or absence of MS-275. The preproinsulin mRNA expression was then measured by quantitative real-time PCR. The mRNA level was normalized against the *Rplp0*/*RPLP0* and the expression level from control cells was set to 100%. The values correspond to the mean ± SEM of 4 independent experiments. *Significantly different from control condition ($p < 0.05$). **b**) Insulin content of MIN6 cells exposed for 48 hr to (filled bars) 0.5 mM palmitate and MS-275. Insulin content from MIN6 cells was quantified by mouse insulin ELISA kit (Mercodia). Data (mean ± SEM) are representative of four independent experiments. *Significantly different from control condition ($p < 0.001$). **c**) Assessment of glucose-induced insulin secretion in MIN6 cells cultured with palmitate and MS-275 for 48 hrs. For measurement of glucose-induced insulin secretion, the cells were pre-incubated for 60 min in Krebs-Ringer buffer (KRB) containing 2 mmol/l glucose (basal) and successively incubated either in the same buffer or in KRB containing 20 mmol/l glucose (stimulated). The amount of insulin release and content was quantified by mouse insulin ELISA kit (Mercodia). The results are expressed as the ratio between the amount of insulin released in the medium under stimulatory and basal conditions over insulin content, and are the mean ± SEM of three independent experiments. (***$p < 0.001$)

a

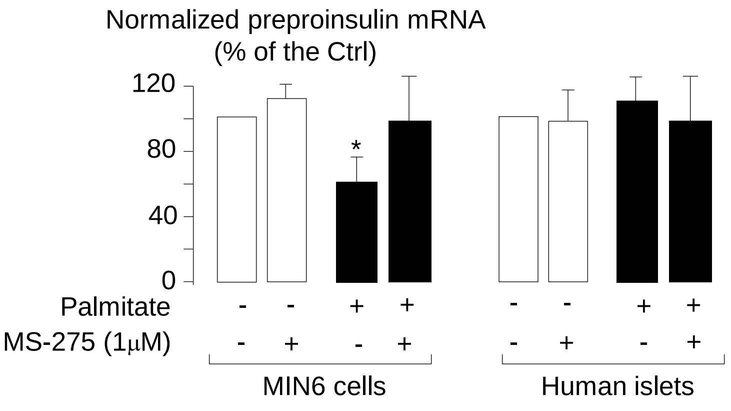

b

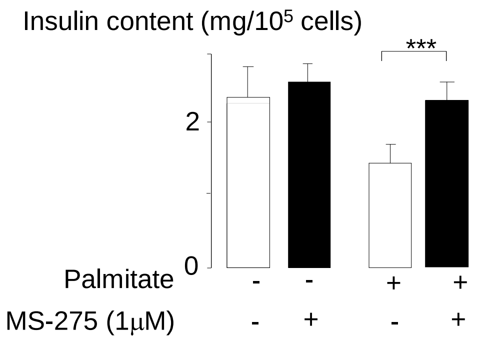

c

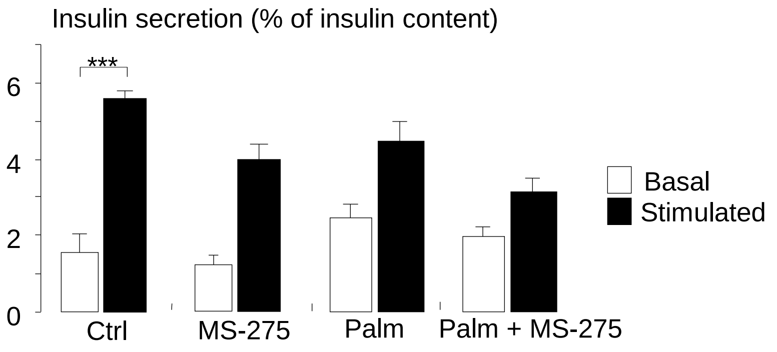

Supplement: Supplementary file 1 — Supplementary Figure 1: Effect of palmitate on the HDACs activity and expression. Supplementary Figure 2: Measurement of preproinsulin mRNA, insulin content and glucose-induced insulin secretion in response to MS-275. [file 195739.f1.pdf]
